# Suicidal ideation and thoughts of self-harm during the COVID-19 pandemic among Swedish employees: a cohort study on the role of job instability and job insecurity

**DOI:** 10.1186/s40359-024-02131-8

**Published:** 2024-11-04

**Authors:** Sandra Blomqvist, Hugo Westerlund, Linda L. Magnusson Hanson

**Affiliations:** Stress Research Institute, Division of Psychobiology and Epidemiology, Department of Psychology, Stockholm University, Albanovägen 12, Stockholm, SE-106 91 Sweden

**Keywords:** COVID-19, Employment insecurity, Organizational change, Restructuring, Staff reduction, Suicidality

## Abstract

**Background:**

Suicidal ideation may be a warning sign for suicide and previous work has indicated a higher prevalence of suicidal ideation during the COVID-19 pandemic. Job loss and job insecurity are potential risk factors for suicidal ideation, but their importance during the pandemic, and the role of organizational changes for suicidal ideation, is unclear. This study examined the association between various experiences associated with job loss and job insecurity during the pandemic and thoughts of suicide/self-harm in Sweden.

**Methods:**

The study sample was drawn from the Swedish Longitudinal Occupational Survey of Health (SLOSH). Auxiliary data collections in February 2021 and 2022 assessed exposure to job loss/unemployment, furlough, workplace downsizing, or increased job insecurity versus stable employment and thoughts of suicide or self-harm (PHQ-9) during the pandemic. The analyses were based on 1558 individuals (2 349 observations) participating in either or both waves and who had been working before the pandemic. Logistic regression models with cluster-robust standard errors were fitted, including sociodemographic factors and prior mental health problems to control for potential confounding. Measures of personality based on a brief version of the Big-Five personality inventory were also added.

**Results:**

The results indicated an association between all experiences, except furlough, and thoughts of suicide/self-harm, when adjusting for sex, age, civil status, socioeconomic status and prior mental health (job loss odds ratio (OR) = 3.70, 95% confidence interval (CI) 1.79–7.63, downsizing OR = 2.41, CI 1.24–4.70, job insecurity OR = 2.77, CI 1.15–6.67). The associations for job loss and insecurity were attenuated by adjustment for personality, although it remained statistically significant for downsizing.

**Conclusions:**

The results suggested a higher risk of suicidal ideation connected with loss of employment and survival of a downsizing, but not a forced reduction in working times/pay during the COVID-19 pandemic. The association for subjective job insecurity was less robust and may be partly explained by personality.

**Supplementary Information:**

The online version contains supplementary material available at 10.1186/s40359-024-02131-8.

## Background

The COVID-19 pandemic resulted in dramatic changes in living and working conditions. A large part of the workforce was forced to adapt their work practices/work arrangements, including working remotely. In addition, many companies were forced to make organizational changes or restructure and/or utilize job retention schemes to cope, especially in certain labor market sectors. Although these changes may have had both negative and positive effects on mental health, a general increase in mental health problems across the pandemic has repeatedly been reported in the general population in high income countries [1, 2] and in some occupational groups such as health care personnel [3, 4]. Concerns about increased suicidality were also raised and some studies have suggested a higher prevalence of suicidal ideation (“thoughts about ending one´s life”) [5], and suicidal behavior (suicide or suicide attempt i.e. “self-injurious behavior with inferred or actual intent to die”) [5] during the pandemic [6, 7], although no rise in suicide has been seen in other studies [8, 9].

Suicide (“intentionally ending one´s own life”) is a significant cause of death globally [10] and it has been recognized that loss of employment and financial stressors are risk factors for suicide [5, 11–13]. Previous work has also linked life events involving job loss and job insecurity with suicidal ideation and/or suicidal behavior [14, 15]. However, relatively little is known about the relationship between job loss, job instability, job insecurity and suicidality during the COVID-19 pandemic [16–18]. Furthermore, many countries scaled up existing short-time work schemes during the pandemic, introduced new ones or created temporary wage subsidies to preserve jobs. Some scholars have concluded that such job retention schemes at least initially contributed to protect employees from poor mental health during the pandemic [19] and this is in line with the findings of a previous study of ours among Swedish workers that found no increased risk for anxiety and depression among furloughed workers [20]. However, no previous study has to our knowledge investigated the association between furlough and suicidal thoughts. Studies on organizational changes such as downsizing and suicidality including suicidal ideation are also lacking.

Although the development of suicide risk is complex, suicidal ideation warrants particular attention in research. Suicidal ideation normally precedes suicidal behaviors [21] and may be followed by an attempt within a relatively short amount of time, such as within 12 months of the onset of ideation [22]. Moreover, it has been recognized that factors that contribute to suicidal ideation are likely to be different from factors of relevance for progression from ideation to attempt or suicide [5]. Hence, identification of and intervention targeting risk factors specifically for suicidal ideation, and consideration of such risk factors in clinical practice, could significantly impact suicide risk.

This study aimed to examine the association between experiences of job loss, workplace downsizing, furlough, and perceptions of job insecurity during the COVID-19 pandemic in Sweden and thoughts of suicide or self-harm.

## Materials and methods

### Study population/sample

Until the year 2020, the Swedish Longitudinal Occupational Survey of Health (SLOSH) cohort comprised 40,877 Swedish men and women originally participating in the Swedish Work Environment Surveys (SWES) 2003–2011. These men and women were 16–64 years of age at baseline, in paid work and representative of various occupations nationwide. These participants have been followed up through self-completion questionnaires every second year beginning in 2006, 2008, 2010 or 2014 [23]. In total, 30,478 (75%) of those individuals had responded to follow-up questionnaires at least once until 2020, with response rates varying between 65% and 48% over the years. Non-responders to follow-up questionnaires were included in future data collections unless they died, emigrated or actively opted out. Both retrospective and prospective data from a number of administrative registers are also collected.

The present study was based on a subsample from SLOSH 2020, contacted for the “SLOSH-corona” study. SLOSH-corona collected auxiliary data in the context of the COVID-19 pandemic. Out of all respondents in the SLOSH 2020 data collection (*n* = 17 489), 3 041 agreed to be contacted, provided correct contact details and agreed to receive an invitation to a web-based survey via Survey & Report. Of these, 1 902 responded to the SLOSH-corona web survey in Jan-Feb 2021 (SLOSH corona 1), and the majority (*n* = 1 580) also responded to the follow up survey one year later (SLOSH corona 2), to which an additional 700 individuals responded to. In the present study, we included only those respondents reporting that they were working prior to the COVID-19 pandemic (*n* = 1 680), who had not retired and provided information about the key measures of job loss/instability and insecurity, and mental health. This resulted in an analytical sample of 1 558 individuals and a total of 2 349 observations. Analyses indicated that individuals in this subsample differed to some extent from all other respondents to SLOSH 2020 (Supplementary Table 1). We found a higher proportion with university education and non-manual socioeconomic status among individuals in our analytic sample reporting to be in paid work at least 30% of full time during the past 3 months than among all other participants in SLOSH 2020 (data collected April-September) reporting to be in paid work at least 30% of full time during the past 3 months. Our analytic sample also tended to consist of a slightly higher proportion of women.

## Main measure of job loss, job instability, and job insecurity

The exposure of interest comprised job loss, job instability/organizational changes, including downsizing and furlough, and job insecurity across two different study periods; from March 2020 (start of the pandemic) to February/March 2021 and from February/March 2021 to February/March 2022 (when most restrictions were beginning to be phased out). Measures of exposure for each period were created with mutually exclusively ordered categories, considering the continuum from job loss (becoming unemployed or dismissed), furlough (forced reduction in working time and pay without loss of employment), downsizing (working at a workplace with staff reduction), and job insecurity to stable and secure employment. If the participants had been exposed to several of the phenomena in this continuum, they were classified according to the foregoing of those phenomena. This meant that a participant who had experienced furlough was classified as exposed to furlough only if not experiencing job loss in the same study period. Similarly, a participant was classified as exposed to downsizing only if he/she had not experienced job loss or furlough during the same period. A few of the participants who had been noticed were also included in the downsizing category since they were still employed at the workplace. Moreover, those classified with job insecurity had not experienced job loss or instability but had a subjective perception of reduced job security. Those who did not experience any of these phenomena, but otherwise reported working prior to the pandemic and were still in the workforce (e.g. had not gone directly from employment to retirement during the pandemic), were considered as the reference group. More information about the questionnaire items can be found in the appendix (Additional file 1).

## Measure of thoughts of suicide or self-harm

The web survey also included questions about mood and feelings during the past 2 weeks according to the Patient Health Questionnaire 9 items (PHQ-9) [24, 25]. This instrument has shown high reliability, good diagnostic criterion and construct validity for depression [25]. The instrument includes one item about “thoughts that you would be better off dead or of hurting yourself in some way” which was used as the outcome variable in the study. The respondents were categorized as having thoughts of suicide or self-harm if they reported such troubles several days, more than half of the days or nearly every day over the past 2 weeks.

## Covariates

Register-based age, sex, and socioeconomic classification (manual or non-manual occupations based on a Swedish socioeconomic classification system), complemented by self-reported information on civil status (married/cohabiting or single) in 2020 were considered as possible confounders. Also, educational level, personal and household income recorded in registers at Statistics Swedish were considered, but since those variables were not associated with the exposure measure or the outcome measure, they were not inlcuded in the final analyses.

For the analyses we also considered presence of anxiety symptoms prior to the pandemic measured by SCL-ANX4, a sub scale to the Symptom Checklist-25 [26, 27], available in SLOSH 2018. Sum scores ranged between 0 and 16 and possible cases with anxiety prior to the pandemic were defined based on scores of ≥ 6 [26]. Similarly, presence of depressive symptoms prior to the pandemic were assessed by means of the SCL-CD6 scale in SLOSH 2018 and cases with depressive symptoms as those with scores above 16 on a sum scale (0–24) [28].

Finally, the personality traits extraversion, agreeableness, conscientiousness, neuroticism and openness were assessed using a brief Big Five personality inventory consisting of 10 questions (BFI-10) [29]. These personality traits were additionally considered in the analysis since certain traits such as neuroticism have been associated with stressors such as job insecurity [30] and heightened risk for mental health problems [31–33] People with high levels of neuroticism may also have elevated risk of emotional problems in the context of stressful experiences [33, 34].

## Statistical analyses

We used logistic regression models to estimate associations with thoughts of suicide or self-harm. A logistic regression model is usually expressed as log (p/(1-p)=β_0_ + β_1_ × _1_ + β_2_ × _2_…+β_k_x_k_, where p=probability of that the outcome is 1, x_1_ to x_k_ is a set of predictors, β_0_ is an interecept and β_1_ to β_k_ are the regression coefficients for the predictors. The logistic regression models were applied with cluster-robust standard errors to derive valid 95% Confidence Intervals (95% CI) around odds ratio estimates (OR) of association. Cluster-robust standard errors, based on cluster-level residuals, were used to account for the dependent nature of the repeated observations within individuals [35]. As main analyses we present (1) crude models, (2) models controlled for sociodemographic factors, (3) models additionally accounting for prior mental health problems, and (4) models additionally considering personality traits. The main analysis relies on categorization of exposure from the first period of the pandemic for those only partaking in the first survey, from both periods for those partaking in both surveys, and from the second period for those only partaking in the second survey. As the preliminary analyses including time of data collection gave the same results as analyses without taking time of data collection into account, model results were presented without adjustment for time of data collection.

We further investigated effect modification by sex. This was done by running stratified analyses for men and women, and by including interaction terms in the logistic models. The models with an interaction term were then compared to a simpler model without any interaction using the likelihood ratio test (significance level 0.05). Moreover, Relative excess risk of interaction (RERI) was calculated as an indicator of interaction on the additive scale. However, the power was too limited for examining effect modification by neuroticism. Finally, we repeated the main analyses with stabilized propensity scores as weights in the analyses to limit potential selection bias due to non-response [36]. Propensity scores were calculated considering age, sex, birth country, marital status, educational level, income, region (larger cities, other regions with ≥ 50 000 inhabitants, other regions with < 50 000 inhabitants) and subcohort (originally participating in SWES 2003, 2005, 2007, 2009 or 2011) as indicators in the propensity score models, based on 35,742 SLOSH cohort participants. The latter excluded 5115 individuals from the total cohort who had died, emigrated, opted out of the study or unable to participate for other reasons in 2020. All analyses were performed using SAS 9.4 with proc surveylogistic with specified cluster id.

## Results

Table 1 presents statistics on exposure, outcome, sociodemographic characteristics, and prior mental health problems. The statistics include number of observations and are presented according to year of data collection. The statistics show that job loss, instability, and especially furlough, were more common in the earlier part of the coronavirus pandemic (March 2020-beginning of 2021) than in the later part (beginning of 2021-beginning of 2022). While 11% experienced furlough until the beginning of 2021, only 4% reported furlough between beginning of 2021 and 2022. The sample consisted of a higher percentage of women than men, a relatively high proportion of people over 50 years of age and a high proportion of married or cohabiting and Swedish born participants. Overall, 4% reported thoughts of suicide or self-harm.

**Table 1:** Sample characteristics (all 2349 observations)

		2021 (*n* = 1100)		2022 (*n* = 1249)	
		*n* (mean)	% (sd)	*n* (mean)	% (sd)
Exposure to job loss/instability/insecurity	Stably employed	795	72	1012	81
	Increased job insecurity	28	3	44	4
	Downsizing	97	9	100	8
	Furlough	120	11	46	4
	Job loss/unemployment	60	5	47	4
Outcome	Thoughts of suicide or self-harm	43	4	48	4
Sex	Women	620	56	760	61
Age	Average age	54.9	11	54.6	11
Civil status	Married/cohabiting	845	77	971	79
Socioeconomic status	Non manual	893	82	1035	84
Birth country	Born in Sweden	1042	95	1182	95
Prior mental health problems	Symptoms of depression 2018	37	3	45	4
	Symptoms of anxiety 2018	133	12	160	15

Regarding job loss/unemployment, the logistic regression analyses suggested an association with thoughts of suicide or self-harm (Table 2). The odds ratio (OR) with 95% confidence interval (CI) was 3.70, 1.79–7.63 when adjusting for sex, age, civil status and manual or non-manual socioeconomic status. Moreover, those who had been employed at a workplace with staff reductions and those who had experienced increased job insecurity without job loss appeared to have an excess risk of thoughts of suicide or self-harm in these analyses (OR for downsizing 2.41; 1.34–4.70 and OR for job insecurity 2.77; 1.15–6.67). However, the analyses indicated no excess risk associated with furlough (OR 0.76; 0.27–2.12). The results were relatively similar when prior mental health problems were additionally taken into account. Partly different results were, however, obtained when accounting for personality traits in addition to sociodemographic characteristics. The OR for furlough remained statistically non-significant (0.87; 0.23–3.28) and the OR for downsizing remained statistically significant (2.28; 1.05–4.96). The OR for job loss/unemployment, however, increased (4.78; 1.92–11.93), while the OR for job insecurity was markedly attenuated and was no longer statistically significant (to 1.97; 0.61–6.30) when taking personality traits into account.

**Table 2:** Associations between job instability/insecurity during the COVID-19 pandemic and thoughts of suicide/self-harm

	Model 0			Model 1^2^			Model 2^3^			Model 3^4^		
	OR^5^	95% CI l^6^	95% CI u^7^	OR^5^	95% CI l^6^	95% CI u^7^	OR^5^	95% CI l^6^	95% CI u^7^	OR^5^	95% CI l^6^	95% CI u^7^
Stably employed	ref			ref			ref			ref		
Increased job insecurity	**2.88**	**1.20**	**6.92**	**2.77**	**1.15**	**6.67**	**2.75**	**1.02**	**7.45**	1.97	0.61	6.30
Downsizing	**2.47**	**1.27**	**4.78**	**2.41**	**1.24**	**4.70**	**2.20**	**1.06**	**4.59**	**2.28**	**1.05**	**4.96**
Furlough	0.80	0.28	2.24	0.76	0.27	2.12	0.67	0.20	2.23	0.87	0.23	3.28
Job loss/unemployment	**4.05**	**2.01**	**8.15**	**3.70**	**1.79**	**7.63**	**3.89**	**1.75**	**8.65**	**4.78**	**1.92**	**11.93**

^1^ Model 0 crude model i.e. unadjusted for covariates

^2^ Model 1-sex, age, manual/non-manual, married/cohabiting or single

^3^ Model 2-model 1 plus prior poor mental health problems

^4^ Model 3-model 1 plus big 5 personality traits

^5^ OR = Odds ratio

^6^ CI l = Confidence interval lower bound

^7^ CI u = Confidence interval upper bound

Among the dimensions of the Big-5 personality traits, the ORs for thoughts of suicide/self-harm were increased for neuroticism, decreased for agreeableness, while no association with extraversion, conscientiousness, openness to experience were observed (Supplementary Table 2). The analyses also indicated a more marked attenuation of the job insecurity risk estimate when considering neuroticism than some of the other personality traits (Supplementary Table 3). The OR for job insecurity for instance decreased from 2.77 (1.15–6.67) to 1.96 (0.76–5.06) when neuroticism was added to Model 1, while it decreased to 2.52 (1.26–5.04) when the extraversion was added separately to Model 1. Agreebleness also attenuated the job insecurity estimate markedly (from 2.77; 1.15–6.67 to 2.04; 0.80–5.24). Moreover, the OR for downsizing decreased from 2.41 (1.24–4.70) to 2.19 (1.09–4.41) when neuroticism was added to Model 1, while it remained similar or increased when the other traits were added separately to Model 1. For job loss/unemployment, however, more marked attenuation of the risk estimates was observed when adjusting for conscientiousness than when adjusting separately for some of the other personality traits (extraversion, agreeableness, neuroticism).

There were no indications of an interaction between the exposure categories and sex nor between job loss, downsizing and job insecurity on the one hand and neuroticism on the other hand (Supplementary Table 4).

In sensitivity analysis using stabilized propensity scores as weights, adjusting for sex, age, manual/non-manual working status and civil status, the findings were relatively similar to the main analyses (Supplementary Table 5). The OR estimating the risk for thoughts of suicide/self-harm was slightly higher for job loss/unemployment, while it was slightly lower for downsizing and increased job insecurity. The estimate for job insecurity was not statistically significantly increased in the sensitivity analyses, but there was still a tendendy towards excess risk for thoughts of suicide/self-harm.

## Discussion

The present study suggests that not only those who became unemployed in Sweden during the COVID-19 pandemic had an elevated risk of suicidal thoughts, but also survivors of a downsizing, i.e., those who continued to work at workplaces with staff layoffs. Increased job insecurity in a context without downsizing also appeared to be associated with suicidal ideation but this association was potentially explained by personality traits. Finally, no elevated risk was observed for furlough.

The results of this study support previous findings regarding job loss and suicidal behavior [37–39]. A relationship between job loss and suicidal ideation during the pandemic has also previously been indicated [16, 17] and between job loss and suicidal thoughts and suicidal behaviors [18]. Prior studies on downsizing have furthermore found indications of mental health consequences [40]. No previous study has, however, to our knowledge, examined the relationship between downsizing and suicidal behavior or ideation, although prior work on aggregated data suggest that economic crises raise suicide rates [12, 41–43]. Our study therefore adds to the literature on downsizing and suicidality and is in line with earlier findings concerning job insecurity. Even without job loss, survivors of a downsizing may be at increased risk of mental health problems due to increased job insecurity, which has been previously associated with both suicidal ideation [14] and suicide [15]. Moreover, in a repeated cross-sectional study, a link between worry about job loss and suicidal ideation during the pandemic was found [44]. With regard to job insecurity, however, this study examines people with a perception of reduced job insecurity without job loss, and job instability. This measure is likely to capture largely subjective job insecurity (especially fear or worry of job loss) [45] rather than “objective” job insecurity including experiences or threats of job loss and loss of income and other material or social benefits. This kind of subjective measure may be more strongly related to health outcomes [46] but also personality traits or vulnerabilities such as neuroticism and negative affectivity. Since neuroticism may be a risk factor for both job stressors and suicidal ideation it may be a confounder for the association between job loss/unemployment, job instability/insecurity. Given that the risk estimate for job insecurity was markedly reduced when adjusting for neuroticism, the study appears to support the view of neuroticism as a confounder. However, neuroticism may alternatively be an effect modifier of the association, which could not be assessed in our study. Moreover, our analyses were based on relatively few subjects and we cannot rule out that our measure of job insecurity is associated with an increased risk even after adjustment for personality traits. It should also be acknowledged that measures of personality traits may not be completely stable over time and may be influenced by job insecurity [30]. It is therefore possible that neuroticism functions a mediator of the relationship of interest which would mean that the association of interest is underestimated when adjusting for personality traits. Hence, further research on this topic is warranted.

Furlough may also be expected to contribute to job insecurity. However, even if a furlough during the pandemic may have been involuntary, it could also have been positive for some workers since they avoided job loss, had a reduced risk of COVID-19 infection, and more spare time. Furthermore, whether an experience of furlough is perceived as stressful or not may depend on financial strain [47], and since the financial circumstances during the pandemic were relatively good due to relatively generous short-time benefits in Sweden [48], perceptions of job insecurity and stress may have been limited. During the first and parts of the second wave of the pandemic, for example, a work time reduction of 80% was accompanied by a pay cut of merely 12% in Sweden. Few studies on furlough during the pandemic are thus far available, but our findings seem to be generally in line with findings from the UK showing that furloughed workers were not at significantly increased risk of psychological distress compared to persons remaining in work [19]. A study from South Africa found mental health benefits if the employees were on paid leave while there were no mental health benefits to being furlough without pay [49]. In another study, Abrams et al. (2022) found that older workers were at increased risk of depressive symptoms when furloughed (presumably referring to leave of absence) but not when having reduced hours or income [50]. It is possible that these inconsistent findings may be explained by differences in furlough/short-term work schemes. More research thus seems to be needed on mental health and different forms of furlough/short-term work, on possible long-term effects, and the influence of degree and duration to guide future crises of similar character. Although we had data on degree and duration of furlough, the study was, unfortunately, underpowered to examine this in relation to thoughts of suicide/self-harm.

Other limitations of the present study concern the measurements of the outcome and exposures. Firstly, our data were based solely on questionnaires which is usually associated with a risk for common method bias (dependent misclassification of exposure and outcome). A limitation of the outcome measure is that it includes both thoughts of suicide and self-harm. While thoughts of suicide is a clear indicator of suicidal ideation, i.e. plans for suicide or thoughts about wishing to die [5], thoughts of self-harm may refer to self-injurious behavior without an intent to die. It would have been preferable with an independent measure of suicidal ideation, as the consequences and predictors of thoughts of non-suicidal self-harm may differ. Self-harm is on the other hand, also a risk factor for suicide [5] and the PHQ item used in the present study has been found predictive of suicide and suicide attempt [51]. However, far from everyone with suicide thoughts attempt actual suicide. The knowledge about when and for whom suicidal ideation proceeds to an attempt is still limited [52]. It is also known that suicidal thoughts are closely linked to depressive mood. However, suicidal thoughts seem to be the least common symptom qualifying for a diagnosis according to the DSM-V criteria [51], and suicidal thoughts can also exist without mental disorder. The measure of suicidal thoughts therefore only partly overlaps with the PHQ-9 measure of depression. Furthermore, we lacked information about thoughts of suicide or self-harm before the COVID-19 pandemic. With regard to exposure, a limitation is that we did not capture multiple risk factors during the same particular study period. Some of the exposure categories are, however, conceptually and empirically linked and may be part of the same process [53]. Another possible limitation of the exposure measure is that respondents were asked to retrospectively recall and report their experience of job loss, job instability and insecurity. Especially the assessment of job insecurity may thereby be influenced by recall bias, leading to an exaggerated association between job insecurity and thoughts of suicide or self-harm. Adjustment for prior mental health problems may, however, limit the risk for bias. Further adjustment for personality traits may also have limited the risk of common method bias since personality traits such as neuroticism or negative affectivity may influence subjective reports in questionnaires. Additional adjustment for personality traits is therefore a strength of the study. The measures of personality traits were, however, based on limited number of items with poorer psychometric properties than full-length Big Five measures [29]. The generalizability to other countries is questionable as the design of the short-time working schemes varied across countries [54] and the Swedish COVID-19 pandemic strategy differed in certain ways from that of many other countries [55]. Possible self-selection should also be considered when interpreting the findings. It is known that e.g. women, highly educated people, middle-aged and older workers are overrepresented in SLOSH [23] and that an even higher proportion of highly educated choose to participate in SLOSH-corona. This is likely to restrict generalizability of the results to certain groups of people in working age with lower degree of attachment to the labor market including people with precarious work or atypical employments (i.e. irregular, poorly paid, insecure, unprotected employment) [56]. Selection due to e.g. attrition may be associated with selection bias if the association between exposure and outcome differs among those included in the analytic sample and others that could not be included in study. However, selection bias did not appear to be major issue in the present study according to the additional analyses based on stabilized propensity score weights, although these analyses need to be interpreted cautiously as they may not account for all relevant factors for selection and for attrition in earlier phases (participation in the Labor Force survey and subsequently the Swedish Work Environment Survey). The study also has a number of additional strengths including repeated measures, when available. Further, the measure of job loss, instability/insecurity took account of a continuum from job loss to job insecurity [53]. The questions on job loss/unemployment, instability/insecurity also referred to periods temporally prior to the measurement of thoughts of suicide/self-harm decreasing the risk that associations are explained by reverse causation and increases the likelihood that the associations represent cause and effect associations. A range of covariates was adjusted for in our analyses, although we lacked information on potentially relevant factors such as family history early life adversities and other life events [5].

## Conclusions

The results suggested a higher risk of suicidal ideation connected to job loss and survival of a downsizing, but not a forced reduction in working times/pay during the COVID-19 pandemic. The association for subjective job insecurity was less robust and may be partly explained by personality. The study implicates that generous short-time working schemes in times of crisis may lower suicide risk and may guide future efforts to decrease the risk for mental ill-health and its consequences. The study also implies that preventive actions to moderate the impact of downsizing may be important in restructuring processes, also in times of economic stability and lower levels of unemployment. This may include employer involvement of professionals in risk assessments, with knowledge of the complexity of suicidal behavior and suitable responses, and provision of increased employee access to mental health services and treatment [57]. Enhanced training of health care professionals in the potential work stressors that can contribute to suicidal ideation, and consideration of those stressors in clinical assessment, diagnosis and management may also contribute to decrease suicide risk.

## Electronic supplementary material

Below is the link to the electronic supplementary material.

Supplementary Material 1

## Data Availability

Given restrictions from the ethical review board and considering that sensitive personal data are involved, the data that support the findings of this study is not possible to make freely available. Access to the data may be provided to other researchers in line with Swedish law and after consultation with the Stockholm University legal department. Requests for data, stored at the Stress Research Institute, Department of Psychology, Stockholm University, should be sent to the corresponding author or data@slosh.se.

## Abbreviations

SLOSH: The Swedish Longitudinal Occupational Survey of Health
PHQ-9: Patient Health Questionnaire (version consisting of 9 items)
SCL: Symptom Checklist
SCL-ANX4: Symptom Checklist subscale for anxiety (version consisting of 4 items)
SCL-CD6: Symptom Checklist core depression subscale (version consisting of 6 items)
BFI-10: Big Five Inventory (version consisting of 10 items)
OR: Odds ratio
CI: Confidence Interval
RERI: Relative excess risk of interaction
SAS: SAS Statistical software
DSM-V: The Diagnostic and Statistical Manual of Mental Disorders (version 5)
