# Supplementary material for: Suicidal ideation and thoughts of self-harm during the COVID-19 pandemic among Swedish employees: a cohort study on the role of job instability and job insecurity
Source: BMC Psychol. 2024 Nov 4;12:621. doi: 10.1186/s40359-024-02131-8 (PMC11536528; doi:10.1186/s40359-024-02131-8)
Supplement: Supplementary file 1 — Supplementary Material 1 [file 40359_2024_2131_MOESM1_ESM.docx]

**Supplementary Table 1.** Comparison of sample characteristics among individuals included in the analyses in the present study reporting to be in paid work at least 30% of full time during the past 3 months in SLOSH 2020 (data collected April-September) and all other respondents in SLOSH 2020 reporting to be in paid work at least 30% of full time during the past 3 months. Any differences in characteristics between the groups according to chi-square tests or t-tests are also indicated in the table.

|  |  | Individuals included in the study sample and working in SLOSH 2020 (n=1274) | | All other SLOSH respondents 2020 (n=9020) | |
| --- | --- | --- | --- | --- | --- |
|  |  | **n (mean)** | **% (sd)** | **n (mean)** | **% (sd)** |
| **Sex** | Women | 771 | 61* | 5125 | 57* |
| **Age** | Average age | 52 | 9.3 | 53 | 9.0 |
| **Socioeconomic status** | Non-manual | 1065 | 84* | 6458 | 73* |
| **Civil status** | Married/cohabiting | 982 | 78 | 7097 | 79 |
| **Educational level** | High (university education) | 863 | 68* | 5057 | 56* |
| **Birth country** | Born in Sweden | 1235 | 97 | 8661 | 96 |
| **Prior mental health problems** | Symptoms of depression 2018 (scale 0-24) | 5.2 | 4.9 | 5.3 | 5.0 |
|  | Symptoms of anxiety 2018 (scale 0-4) | 1.4 | 1.2 | 1.4 | 1.4 |

*=P-value <0.05 indicating a difference in sociodemographic characteristics between those working in SLOSH 2020 (beginning of the pandemic) and included in our study and all other respondents in SLOSH 2020 who reported working at the time of data collection

**Supplementary Table 2.** Associations between job instability/insecurity during the Covid-19 pandemic, selected covariates and thoughts of suicide/self-harm.

|  | Model 0 | | | Model 1^1^ | | | Model 2^2^ | | | Model 3^3^ | | |
| --- | --- | --- | --- | --- | --- | --- | --- | --- | --- | --- | --- | --- |
|  | OR^4^ | 95% CI l^5^ | 95% CI u^6^ | OR^4^ | 95% CI l^5^ | 95% CI u^6^ | OR^4^ | 95% CI l^5^ | 95% CI u^6^ | OR^4^ | 95% CI l^5^ | 95% CI u^6^ |
| **Stably employed** | ref |  |  | ref |  |  | ref |  |  | ref |  |  |
| **Increased job insecurity** | **2.88** | **1.20** | **6.92** | **2.77** | **1.15** | **6.67** | **2.75** | **1.02** | **7.45** | 1.97 | 0.61 | 6.30 |
| **Downsizing** | **2.47** | **1.27** | **4.78** | **2.41** | **1.24** | **4.70** | **2.20** | **1.06** | **4.59** | **2.28** | **1.05** | **4.96** |
| **Furlough** | 0.80 | 0.28 | 2.24 | 0.76 | 0.27 | 2.12 | 0.67 | 0.20 | 2.23 | 0.87 | 0.23 | 3.28 |
| **Job loss/unemployment** | **4.05** | **2.01** | **8.15** | **3.70** | **1.79** | **7.63** | **3.89** | **1.75** | **8.65** | **4.78** | **1.92** | **11.93** |
| Male (ref=Female) |  |  |  | 1.33 | 0.82 | 2.16 | 1.45 | 0.85 | 2.48 | **1.75** | **1.02** | **3.01** |
| Manual worker (ref=Non-manual worker) |  |  |  | 1.21 | 0.67 | 2.16 | 1.04 | 0.55 | 1.97 | 1.07 | 0.55 | 2.09 |
| Age (continuous) |  |  |  | 0.99 | 0.97 | 1.02 | 1.00 | 0.97 | 1.02 | 1.00 | 0.98 | 1.03 |
| Unmarried/ not cohabiting (ref=Married or cohabiting) |  |  |  | 1.27 | 0.74 | 2.18 | 1.16 | 0.65 | 2.10 | 1.24 | 0.68 | 2.26 |
| Mental health problems in 2018 (ref=below cut-off) |  |  |  |  |  |  | **3.34** | **2.00** | **5.56** |  |  |  |
| Extraversion (mean score) |  |  |  |  |  |  |  |  |  | 1.11 | 0.76 | 1.62 |
| Agreeableness (mean score) |  |  |  |  |  |  |  |  |  | **0.58** | **0.37** | **0.90** |
| Conscientiousness (mean score) |  |  |  |  |  |  |  |  |  | 1.06 | 0.73 | 1.54 |
| Neuroticism (mean score) |  |  |  |  |  |  |  |  |  | **2.10** | **1.57** | **2.81** |
| Openness to Experience (mean score) |  |  |  |  |  |  |  |  |  | 1.34 | 0.99 | 1.83 |

^1^ Model 1-adjustment for sex, age, manual/non-manual, married/cohabiting or single

^2^ Model 2-model 1 plus adjustment for prior poor mental health problems

^3^ Model 3-model 1 plus adjustment for big 5 personality traits

^4^ OR=Odds ratio

^5^ CI l=Confidence interval lower bound

^6^ CI u= Confidence interval upper bound

**Supplementary Table 3.** Associations between job instability/insecurity during the Covid-19 pandemic and thoughts of suicide/self-harm, when considering each of the 5 personality traits separately.

|  | Modell 5^1^ | | | Modell 6^2^ | | | Modell 7^3^ | | | Modell 8^4^ | | | Modell 9^5^ | | |
| --- | --- | --- | --- | --- | --- | --- | --- | --- | --- | --- | --- | --- | --- | --- | --- |
|  | OR^6^ | 95% CI l^7^ | 95% CI u^8^ | OR^6^ | 95% CI l^7^ | 95% CI u^8^ | OR^6^ | 95% CI l^7^ | 95% CI u^8^ | OR^6^ | 95% CI l^7^ | 95% CI u^8^ | OR^6^ | 95% CI l^7^ | 95% CI u^8^ |
| **Stably employed** | ref |  |  | ref |  |  | ref |  |  | ref |  |  | ref |  |  |
| **Increased job insecurity** | 2.20 | 0.85 | 5.70 | 2.04 | 0.80 | 5.24 | 2.39 | 0.93 | 6.14 | 1.96 | 0.76 | 5.06 | 2.46 | 0.94 | 6.44 |
| **Downsizing** | **2.52** | **1.26** | **5.04** | **2.49** | **1.22** | **5.07** | **2.36** | **1.17** | **4.76** | **2.19** | **1.09** | **4.41** | **2.42** | **1.19** | **4.93** |
| **Furlough** | 0.95 | 0.34 | 2.68 | 0.98 | 0.34 | 2.82 | 0.90 | 0.32 | 2.54 | 0.98 | 0.33 | 2.86 | 0.92 | 0.33 | 2.59 |
| **Job loss/unemployment** | **3.35** | **1.40** | **8.01** | **3.35** | **1.43** | **7.81** | **3.09** | **1.29** | **7.44** | **3.52** | **1.51** | **8.18** | **3.14** | **1.34** | **7.35** |

^1^ Model 5-adjustment for sex, age, manual/non-manual, married/cohabiting or single, and mean score on extraversion

^2^ Model 6-adjustment for sex, age, manual/non-manual, married/cohabiting or single, and mean score on agreeableness

^3^ Model 7-adjustment for sex, age, manual/non-manual, married/cohabiting or single, and mean score on conscientiousness

^4^ Model 8-adjustment for sex, age, manual/non-manual, married/cohabiting or single, and mean score on neuroticism

^5^ Model 9-adjustment for sex, age, manual/non-manual, married/cohabiting or single, and mean score on openness

^6^ OR=Odds ratio

^7^ CI l=Confidence interval lower bound

^8^ CI u= Confidence interval upper bound

**Supplementary Table 4.** Associations between job instability/insecurity during the Covid-19 pandemic and thoughts of suicide/self-harm, according to sex of the respondents when adjusting for age, manual/non-manual, married/cohabiting or single. The stratified results are presented along with results from tests for interaction between job instability/insecurity and sex.

|  | **Men** | | | **Women** | | | **Multiplicative interaction^1^** | **Additive interaction^2^** |
| --- | --- | --- | --- | --- | --- | --- | --- | --- |
|  | **OR^3^** | **95% CI l^4^** | **95% CI u^5^** | **OR^3^** | **95% CI l^4^** | **95% CI u^5^** | **P-value** | **P-value** |
| **Stably employed** | ref |  |  | ref |  |  |  |  |
| **Increased job insecurity** | 2.68 | 0.80 | 8.93 | 2.91 | 0.80 | 10.53 | 0.96 | 0.92 |
| **Downsizing** | **2.91** | **1.05** | **8.07** | 2.07 | 0.86 | 4.98 | 0.56 | 0.49 |
| **Furlough** | 1.42 | 0.46 | 4.42 | na.^6^ |  |  | na.^6^ | na. ^6^ |
| **Job loss/unemployment** | **4.95** | **1.63** | **15.02** | **2.74** | **1.01** | **7.40** | 0.41 | 0.34 |

^1^ Multiplicative interaction was tested by including an interaction term between each of the exposure levels (i.e., job insecurity, downsizing and job loss) against the reference group (i.e.,stably employed) and sex (men = ref.). P-values for these terms (exposure×sex) are reported above

^2^ Additive interaction was tested calculating the relative excess risk due to **interaction** (**RERI**) by each of the exposure levels against the reference group and sex. P-values for these terms (exposure×sex) are reported above

^3^ OR=Odds ratio

^4^ CI l=Confidence interval lower bound

^5^ CI u= Confidence interval upper bound

^6^ No observations of exposure to furlough among women

**Supplementary Table 5.** Associations between job instability/insecurity during the Covid-19 pandemic, selected covariates and thoughts of suicide/self-harm, with propensity score weights in the analyses.

|  | Model 1^1^ | | |
| --- | --- | --- | --- |
|  | OR^4^ | 95% CI l^5^ | 95% CI u^6^ |
| Stably employed | ref |  |  |
| Increased job insecurity | 2.24 | 0.90 | 5.57 |
| Downsizing | 2.13 | 1.05 | 4.31 |
| Furlough | 0.49 | 0.16 | 1.50 |
| Job loss/unemployment | 4.61 | 1.84 | 11.57 |
| Male (ref=Female) | 1.32 | 0.76 | 2.28 |
| Manual worker (ref=Non- manual worker) | 1.02 | 0.54 | 1.93 |
| Age (continuous) | 0.98 | 0.96 | 1.01 |
| Unmarried/ not cohabiting (ref=Married or cohabiting) | 1.51 | 0.81 | 2.84 |

^1^ Model 1-adjustment for sex, age, manual/non-manual, married/cohabiting or single
